# The epidemiology of mild cognitive impairment (MCI) and Alzheimer’s disease (AD) in community-living seniors: protocol of the MemoVie cohort study, Luxembourg

**DOI:** 10.1186/1471-2458-12-519

**Published:** 2012-07-12

**Authors:** Magali Perquin, Anne-Marie Schuller, Michel Vaillant, Nico Diederich, Alexandre Bisdorff, Jean-Claude Leners, Marylène D’Incau, Jean-Luc Ludewig, Danielle Hoffmann, Dirk Ulbricht, Stephanie Thoma, René Dondelinger, Paul Heuschling, Sophie Couffignal, Jean-François Dartigues, Marie-Lise Lair

**Affiliations:** 1 Centre d’Etudes en Santé, Centre de Recherche Public (CRP)-Santé, 1A-1B rue Thomas Edison, L-1445, Strassen, Luxembourg; 2 CRP-Santé now Unit of Educational Measurement and Applied Cognitive Science, University of Luxembourg, Luxembourg, Luxembourg; 3 Cognitive Neurorehabilitation and Psychology Unit, Rehazenter, Luxembourg, Luxembourg; 4 Department of Neurology, Centre Hospitalier de Luxembourg (CHL), Luxembourg, Luxembourg; 5 Department of Neurology, Centre Hospitalier Emile Mayrisch (CHEM), Esch-sur-Alzette, Luxembourg; 6 Long term care facilities: ALA, Pontalize, Erpeldange and Ettelbruck, Luxembourg; 7 CRP-Santé now Quest SA, Luxembourg, Luxembourg; 8 Department of Neurology, ZithaKlinik, Luxembourg, Luxembourg; 9 Department of Geriatrics, Centre Hospitalier Emile Mayrisch (CHEM), Dudelange, Luxembourg; 10 Life Sciences Research Unit, University of Luxembourg, Luxembourg, Luxembourg; 11 Inserm U897 Université de Bordeaux II, Bordeaux II, France

**Keywords:** Epidemiology, Population-based study, Nested case-control cohort, Aging, Cognition, Mild cognitive impairment, Alzheimer’s disease

## Abstract

**Background:**

Cognitive impairment and Alzheimer’s disease (AD) are increasingly considered a major public health problem. The MemoVie cohort study aims to investigate the living conditions or risk factors under which the normal cognitive capacities of the senior population in Luxembourg (≥ 65 year-old) evolve (1) to mild cognitive impairment (MCI) – transitory non-clinical stage – and (2) to AD. Identifying MCI and AD predictors undeniably constitutes a challenge in public health in that it would allow interventions which could protect or delay the occurrence of cognitive disorders in elderly people. In addition, the MemoVie study sets out to generate hitherto unavailable data, and a comprehensive view of the elderly population in the country.

**Methods/design:**

The study has been designed with a view to highlighting the prevalence in Luxembourg of MCI and AD in the first step of the survey, conducted among participants selected from a random sample of the general population. A prospective cohort is consequently set up in the second step, and appropriate follow-up of the non-demented participants allows improving the knowledge of the preclinical stage of MCI. Case-control designs are used for cross-sectional or retrospective comparisons between outcomes and biological or clinical factors. To ensure maximal reliability of the information collected, we decided to opt for structured face to face interviews. Besides health status, medical and family history, demographic and socio-cultural information are explored, as well as education, habitat network, social behavior, leisure and physical activities. As multilingualism is expected to challenge the cognitive alterations associated with pathological ageing, it is additionally investigated. Data relative to motor function, including balance, walk, limits of stability, history of falls and accidents are further detailed. Finally, biological examinations, including ApoE genetic polymorphism are carried out. In addition to standard blood parameters, the lipid status of the participants is subsequently determined from the fatty acid profiles in their red blood cells. The study obtained the legal and ethical authorizations.

**Discussion:**

By means of the multidisciplinary MemoVie study, new insights into the onset of cognitive impairment during aging should be put forward, much to the benefit of intervention strategies as a whole.

## Background

Given longer life expectancy along with the lack of efficient therapeutic strategies, cognitive disorders and Alzheimer’s disease (AD) are increasingly considered a major public health problem. The cost of these pathologies represents an important burden for public health policies. Some projections venture a worldwide figure of 81.1 million people affected by dementia by 2040, providing no curative treatment is developed by then [1]. Therefore, better characterization of the preclinical stage of AD has been a crucial challenge for research over the past 10 years. This appears to be the stage at which prevention strategies should prove most efficient in delaying or even avoiding further cognitive decline. From this background emerged the concept of mild cognitive impairment (MCI), advocated by Petersen and co-workers [2]. In spite of the controversy surrounding this concept, it was observed that people who meet the criteria for MCI are likely to progress to dementia at a rate of approximately 12% per year, compared to 1 to 2% for cognitively normal people at the same age [2,3]. The MCI criteria are based on the following decision process: a) A cognitive complaint, preferably corroborated by an informant; b) Essentially normal general cognition, but a deficit in at least one cognitive task considering performances adjusted for age and education level; c) No dementia according to the approved Diagnostic and Statistical Manual of Mental Disorders (DSM) IV criteria; d) Arguments for a cognitive decline, without consequences on daily life activities or with minimal discomfort on accomplishing complex instrumental activities.

Up to now, few studies have explored MCI in healthy people from the general population, and the most recent results have shown the difficulty in approaching MCI prevalence, which obviously vary considerably according the criteria applied [4]. The complexity also lies in the fact that a spontaneous complaint is difficult to estimate [5].

Moreover, no national statistics on AD are available in Luxembourg, let alone on MCI. In line with the EU research program on “medical and social challenges posed by an ageing population and the disabilities associated with old age” (Official Journal of 15 November 2000), the National Fund for Research (FNR), which is the institution that provides financial support to research in Luxembourg, has set up a specific research program. This program aimed to study the epidemiological, psychosocial and biological aspects of the neurodegenerative diseases of old age in the country and to obtain a comparative view of these aspects with the broader European context. The national approach adopted is a holistic one, i.e. multidisciplinary and multidimensional, giving priority to projects which are interactive. The MemoVie study started in 2008, federating scientists, physicians, nurses, neuro-psychologists and statisticians in Luxembourg within a “research consortium”.

## Objectives

Based on an interdisciplinary approach, the MemoVie study has the ambition to consider the problems of cognitive disorders in elderly subjects from different viewpoints. Since no previous overview of these concerns for Luxembourg is available, the first goal of the present study is to provide the national prevalence of subjects suffering from MCI and from AD as well as to identify the “environmental” conditions and biological factors in association with the occurrence of MCI and their evolution to AD.

The “environmental” conditions to be examined are demographic and socio-cultural parameters. Among these socio-cultural parameters, special consideration is given to multilingualism, an aspect of particular importance in Luxembourg since residents practice almost 3 languages on average. Sub-clinical impairments are explored by assessing basic general data, by evaluating cardiovascular risk factors, cardiovascular events, the presence of metabolic disorders (e.g. known and sub-clinical diabetes mellitus), the long-term intake of medications that may impact on cognition and major current comorbidities. Biological factors examined also include the study of APOE4 gene polymorphism, blood protein patterns, and selected blood lipid components. Using non-invasive methods, as well as non-hospital environment, the comprehensive approach proposed in this project should allow studying how these factors and parameters are associated with the MCI stage and its evolution to AD, further exploring the potential interactions that could occur.

## Methods/design

### Study design and sampling frame

Since one of the aims of the MemoVie project is to explore the national prevalence of AD and of MCI, it appears crucial to set up at baseline a representative cohort of the senior population in the country. After stratification by age group and gender, potential participants were randomly selected out of any hospital environment from the national social insurance register covering about 97% of the total population. The General Inspectorate of Social Security or IGSS register helped to construct the initial sampling frame, by selecting Luxembourg residents aged over 64 on January 1st 2008 and providing their address, according to our criteria. These addresses can possibly be those of care homes, when people have already been institutionalized. The participants need to be followed up in order to provide information on the possible evolution from normal cognitive status to MCI and from MCI to AD. Additional spontaneous volunteers (obviously not to be considered in the prevalence study) are concomitantly included in the cohort. Therefore, the cohort is continuously fed in a dynamic way with regular inclusions of new volunteers. The general format of the project is that of a prospective cohort, combined with nested case-control studies. Such a design allows studying specific items within the cohort in accordance with the research question, with no need to conduct these items on all subjects included in the cohort. The nested design strengthens the investigations of the considered outcomes of persons suffering from MCI, AD and cognitively normal people, while reducing the costs of the study.

### Sample size and power estimation

The global format of the study is divided into two parts:

The first one consists in the prevalence study of MCI and AD in Luxembourg considering an expected prevalence of persons with MCI of approximately 20% in the general population aged over 65 or 70 [6-8] and a 2 to 5% prevalence of persons suffering from a sporadic form of AD [9]. Therefore, with a precision of 2.5%, a sample size of 983 is computed with a power of 95% and an alpha risk of 5%. This sample size also offers the opportunity to investigate AD prevalence. The calculation for the latter required a sample size of 753 with a precision of 1%, a power of 95% and an alpha risk of 5%. Assuming 40% of refusal to participate, the sample size amounts to 1377 persons. In addition, this first prevalence study allows setting up the MemoVie cohort.

At the end of the prevalence evaluation, the global sample is expected to be constituted with groups of participants defined as “cognitively normal”, “MCI” and “persons with probable AD” in frequencies of about 780, 200 and 20 respectively. At this stage, comparison is drawn in a case-control assessment between the groups according to the different parameters. Power calculations show that a group of 200 MCI subjects and 200 control subjects yields statistically significant odds ratio of 2.3 (alpha error 5% and 80% power).

**Figure 1:**
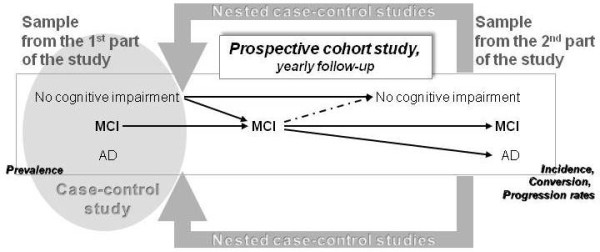
**Conduct of the study.** The sample resulting from the 1st part of the study should be at least composed of cognitively normal people, MCI and AD (grey circle). This sample should allow the prevalence and case-control studies. Arrows depict the different situations/ evolutions occurring over time. The dotted line shows a potential critical situation due to wrong classification of MCI. The sample resulting from the 2nd part of the study should offer the opportunity to obtain the incidence of MCI and/or AD as well as the progression rate between stages.

The second part of the project consists in the prospective cohort of people aged over 64 years who presented normal cognition or MCI. Participants with AD at baseline are not further followed up. The cohort leads to comparative analysis between the main outcomes, i.e. evolution from normal cognitive capacities to MCI and evolution from MCI to AD (Figure 1). We adopt as initial (pessimistic) assumption 12% of MCI subjects likely to progress to AD in the course of 3 years. A studied characteristic present in 25% of MCI subjects is supposed to be associated with a 30% chance of 3-year progression to AD. Then, given the 200 MCI subjects, the statistical significance of a relative risk of 3.0 could be shown in this study.Figure 1Constitution of the MemoVie cohortConstitution of the MemoVie cohort. The sample resulting from the 1st part of the study should be at least composed of cognitively normal people, MCI and AD (grey circle). This sample should allow the prevalence and case-control studies. Arrows depict the different situations/evolutions occurring over time. The dotted line shows a potential critical situation due to wrong classification of MCI. The sample resulting from the 2nd part of the study should offer the opportunity to obtain the incidence of MCI and/or AD as well as the progression rate between stages.

### Recruitment procedure

Before sending individual invitation letters, several strategies are used to enhance participation in the project: 1) The study is advertised in the local newspapers in order to inform the general population, and thereby create awareness; 2) All physicians whose practice could mainly target the elderly (general practitioners, internists, neurologists, psychiatrists and physicians specialized in reeducation and functional rehabilitation) received a letter and a USB key containing a personalized letter with a description of our approach, the summary of the project, the composition of its research team, its partners and the information note accompanying the informed consent given to participants. The purpose was to inform health professionals about our study, its objectives and issues, in case some of their patients were selected and wished to discuss the project and collect their views.

The inclusion criteria imposed at baseline are: to reside in Luxembourg and to be aged at least 65 years. Exclusion criteria are the conditions that may prevent the neuropsychological evaluation i.e. non- or poorly sighted, and people speaking none of the proposed languages: i.e. official and practiced languages in the country such as Luxembourgish, French, German, English, Portuguese and Italian.

The recruited seniors are sent a letter of invitation that described the study and set out its purpose and scope. Invited persons are given the opportunity to accept or decline participation by returning the reply coupon in a prepaid envelope. One week after the mailing, subjects who have not mailed back the reply coupon, as well as those who have agreed to participate, receive a telephone call from a trained assistant. The telephone interview aims at describing the study one more time, giving people the opportunity to ask questions, stating the invitation as well as scheduling an appointment.

### Evaluation procedure

**Figure 2:**
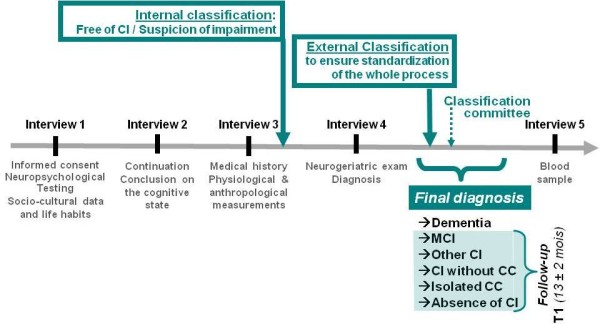
**Conduct of the study.** CC: cognitive complaint, CI: cognitive impairment.

The MemoVie project is based on the following overall procedure of evaluation (Figure 2):

The first interview (of about one hour) with a psychologist specifically trained for this study, during which are performed: 1) another exhaustive explanation of the study before the presentation of the informed consent form for signature, 2) the collection of information on the social and cultural lifestyle of the participant, 3) the first part of the neuropsychological tests (see Neuropsychological evaluation).

The second interview with the same psychologist (± one hour), performed more than 1 week after the first one, and less than 6 months from it, during which: 1) the informed consent form is signed when the participant asked for a reflection period at the first interview, 2) the neuropsychological tests are completed, 3) an evaluation of linguistic abilities is performed.

The third interview (± 45 minutes), performed by a trained research nurse, during which participants undergo a health evaluation and risk factor assessment (including physiological and anthropological measurements, see Research nurse evaluation).

After these three interviews, participants are either suspected of cognitive impairment (CI) or free of cognitive disorders (see Internal classification committee). All the participants suspected of CI and a matched^a^ control group then meet one of the 6 physicians participating in this project. Thus, a second phase of the protocol includes, for some participants only:

A fourth interview (30 to 45 minutes) with a neurologist or a geriatrician, specifically trained for this study, who can rely on the neuropsychological findings and all the observations from the nurse. This neurogeriatric examination allows completing the medical anamnesis and providing the final diagnosis (see External classification). Various tests are also conducted, mostly to assess the mobility of participants in walking and balance, but also their risk of falling (see Neurogeriatric assessment). Gait and balance assessment may indeed augment the diagnostic evaluation of dementia [10].

A fifth meeting between the participants and the trained research nurse (20 minutes) allows closing the protocol with a blood sample collection and an evaluation of the olfactory function, the performance of which decreases in the earliest signs of age-related neurodegenerative disorders [11].

### Site of investigation

The in-person evaluation is proposed and carried out: 1) as far as possible in a “neutral” place (the Centre for Clinical and Epidemiological Investigation, CIEC, CRP-Santé ,Luxembourg) in order to minimize possible environmental interferences; or 2) at home, taking care to specify the necessary optimal conditions (quietness, minimized disturbance). The choice of the place is left to the participants, with the aim of not excluding people who prefer to remain at home or who experience difficulties in moving. The various institutions hosting some participants (homes, integrated centers for the elderly, nursing homes, etc.) constitute another type of investigation site. Thus, psychologists and the nurse, as well as the two geriatricians who are jointly responsible for the appointments at home, visit these places. The four other neurologists participating in the study perform their interviews at the CIEC.

### Neuropsychological evaluation

The psychologist collects demographic information including date and place of birth, marital and parental status. Socio-cultural information is explored, as well as education and professional activity, habitat network, social behavior, leisure, physical activities, history of falls and accidents. Subjects are questioned about their previous consultations with a neurologist or a psychiatrist. The multilingualism ability of the participants is quantified through the number of languages practiced fluently, the age at which the languages were learned, the duration of the actual practice, the rate of use of each language: at home, in daily activities outside (i.e. shops, restaurants, leisure, administrative procedures, transport) and at work. With her agreement, we adapted to our own purposes the Language and Social Background Questionnaire [12] kindly provided by Prof. E. Bialystok.

*The neuropsychological tools chosen* for this study aim at approaching cognitive disorders specific to dementia, especially AD. Additional tests (1) assess CI more specifically associated with MCI conditions, (2) measure potential psychological states interfering with cognitive performance, such as depression and anxiety, (3) estimate cognitive complaints and decline from auto- and hetero-evaluation, and (4) finally appreciate repercussions of the observed deficits on daily activities.

The testing proposed consists in The Consortium to Establish a Register for Alzheimer’s Disease-Neuropsychological Battery (CERAD-NP) and meets all these criteria [13]. We used the extended version (CERAD-NP-plus, see Table 1 for recapitulation) which:

A. measures a large variety of cognitive functions especially episodic memory and attention/executive functions that are considered among the first to be altered in AD [16-18], as well as language and spatial functions, known to be impaired in early dementia as well (DSM IV criteria [19]), implying cognitive deficits in addition to memory impairment: subtests Word List Learning, delayed Word List Recall, Word List Recognition, Visual Memory Test, Trail Making Test A & B, Verbal Fluency (phonological, S) in an acceptable time frame (30-45 minutes); providing valuable information for screening and diagnosis of MCI and early AD if interpreted properly [20];

B. is sensitive to MCI assessment [21, 22];

C. is sensitive to Alzheimer dementia and other forms of dementia [23];

D. is available in the different languages [24] required for the project i.e. the different languages spoken in Luxembourg (2 official languages -French and German- and 3 languages commonly used, Italian, Portuguese and English; http://cerad.mc.duke.edu/ or http://www.memoryclinic.ch);

E. has no global score, the subtests are scored individually, which allows distinct interpretation of each cognitive function;

F. has a decoding program that is easy and fast to use.

G. In addition to being normalized on patients with AD (n = 1,094), the Cerad-NP-plus is normalized on healthy controls (n = 463) [24-26]. It is also classified for different age groups.

**Table 1:** Summary of the tools used for the neuropsychological evaluation

**CERAD-NP:**	
	Verbal semantic fluency (animals)
	Modified Boston Naming Test [14]: denomination test from simple line drawings
	Mini Mental State Exam (MMSE) [15]
	Word List Memory (progressive learning of a 10-item list, direct recall, learning performance on 3 consecutive recalls, delayed recall, intrusions)
	Constructional Praxis
	Word List Recall
	Word List Recognition
	Recall of Constructional Praxis
**Additional tests in the CERAD-plus:**	
	Verbal phonological fluency
	Trail Making Test (TMT A&B; mental flexibility assessment)
**Supplementary tests of the MemoVie protocol**	
	Letter Cancellation D2 Test
	Letter Cancellation D2 Test
	RL/RI-16
	Geriatric depression Scale (GDS)
	Beck Anxiety Inventory (BAI)
	Cognitive Difficulties Scale (CDS)
	Clock-Drawing Test (CDT)
	Frontal Assessment Battery (FAB)
	Informant Questionnaire on Cognitive Decline in the elderly (IQ-Code)
	Questionnaire of cognitive complaints (QPC)

Moreover, remaining within reasonable time for an interview, we chose to enrich the cognitive evaluation by using 9 additional tests added to the CERAD-NP-plus:

H. The free and cued recall of the Grober & Buschke procedure, RL/RI-16 (Rappel Libre/Rappel Indicé à 16 items) which is an episodic verbal memory test [27], with high sensibility in early dementia; it has been recently proposed as core feature in diagnosing AD [28];

I. The Frontal Assessment Battery (FAB) which has been used to explore executive functions in dementias with a frontal dysexecutive phenotype. This test has validity in differentiating fronto-temporal type of dementia from AD in mildly demented patients [29-31];

J. The letter-cancellation test D2 [32] which has been shown to be an independent predictor of conversion from MCI to AD [33];

K. The Clock-Drawing Test (CDT) which is one of a set of brief objective measurements included in the clinical battery to facilitate the clinical diagnosis of AD without reliance on the neuropsychological test results, thereby allowing independent evaluation of the neuropsychological battery [24];

L. The Cognitive Difficulties Scale (CDS) [34] and the questionnaire of cognitive complaints (Questionnaire de Plainte Cognitive, QPC [35]) which allow the psychologist to estimate cognitive complaints and decline by auto-evaluation;

M. The short form of the Informant Questionnaire on Cognitive Decline in the elderly (IQ-CODE, [36]) which is administered to a close person designated by the participant (informant), in order to obtain a hetero-evaluation of the cognitive decline for this participant [37].

N. The Beck Anxiety Inventory (BAI, [38]) and the Geriatric Depression Scale (GDS, [39]) which assess current or previous anxiety and depression.

### Research nurse evaluation

A single nurse performs the following overall evaluation for all the participants. The nurse collects data on major events in the medical history of the participant and personal clinical health status (especially chronic diseases: diabetes, hypertension, cardiovascular and traumatic antecedents, cancer, depression, quality of sleep etc.), together with specific aspects of the family’s medical history. The subjects also report cigarette smoking or tobacco use, alcohol intake, potential xenobiotic exposure and overall well-being (perception of health compared to other individuals of the same age). The name and dose of all the current medications and long-term prescriptions are also collected (subjects are instructed to fill in a table at home, or to bring boxes of current medications for the appointment).

The degree of autonomy is assessed for the essential activities of daily living, measured on three levels: “independence”, “partial need of aid” and “dependency”, according to the ADL [40]. The degree of autonomy in the completion of instrumental activities of daily living is additionally informed according to the IADL scale [41].

During the same interview, systolic and diastolic blood pressure (mmHg) as well as heart rate (beats per minute) are measured three times using a standard sphygmomanometer (Medisana®). Possible orthostatic hypotension is evaluated. Standing body height (cm) is measured using a stadiometer (Seca® 214). Weight (kg) is evaluated with an electronic scale (Seca® 813). Other anthropometric measurements are evaluated in accordance with good practices [42], such as waist, hip, arm, head and calf circumferences. Circumferences of members are measured (cm) using a seam tape about 1.8 cm wide.

In the second phase, the nurse collects a fasting blood sample from the antecubital vein of people suspected to present CI and of those in the control group (see Evaluation procedure). The samples are transported at 10 ± 2°C, (1) in less than one hour to the *Laboratoire de Biochimie* of the Centre Hospitalier de Luxembourg (CHL) and (2) in less than 3 hours, to the *Life Sciences Research Unit* of the University of Luxembourg, before being prepared for later analysis and/or frozen, at -20°C. Concomitantly, the nurse explores hearing and visual deficiencies with a set of questions and administers the Brief Smell Identification Test^TM^ (B-SIT, Sensonics Inc.) to perform a smelling evaluation [43,44].

### Internal classification

After the first three interviews, the internal classification committee, constituted of several members from the research group, meets and discusses a preliminary impression based on the input of each evaluator (the psychologist and the nurse). They indeed previously give their own impression on the cognitive status and the overall health status of the subjects, considering the possible physiological disturbances observed. The committee consequently draws a preliminary cognitive status. Data collected from the CERAD-NP-plus battery at baseline or follow-up are transformed to z-scores of the normative sample of 1,100 cognitively healthy persons [13]. The z-score indicates how many standard deviations an individual person’s cognitive score is from the mean of the normative sample. When z-score > -1.5, persons are judged to have no CI and are enrolled as “cognitively normal subjects”. The classification criteria for suspicion of impairment are: deficient participant performance (i.e. z-score < -2) in ≥ 1 neuropsychological subtest of the overall battery; or poor participant performance (i.e. -2 < z-score < -1.5) in ≥ 2 neuropsychological subtests; or poor in one subtest but with objectified cognitive complaint or psychological symptoms.

Subjects classified positive for suspicion of cognitive or psychiatric impairment as well as the matched control group (see Evaluation procedure) are further assessed by a physician (see Neurogeriatric assessment).

### Neurogeriatric assessment

The neurogeriatric examination is performed by neurologists or by geriatricians who have been specifically trained for the study. The physicians administer the Unified Parkinson’s Disease Rating Scale [45-47], the Timed Get-Up and Go test [48], as well as the Tinetti questionnaire on balance and falls [49-51]. With an ad-hoc manufactured device in line with Newton’s work [52], the physicians administer to participants the Multi-Directional Reach Test, a reliable and valid screening tool for measuring the limits of stability in four directions.

Furthermore, the doctors document their findings about (1) the cognitive complaint of the participant and his/her family (when one of its members had accompanied the participant), (2) the decline reported by the participant him/herself and his/her family (idem), (3) the potential impact on activities of daily living related to cognitive decline, (4) memory and / or other disorders. Finally, based on the data collected by the nurse and the psychologist as a whole, as well as their own clinical impression, the physicians draw conclusions on the cognitive state i.e. if not normal: suspected MCI; suspicion of other disorders (documented) that can affect cognition, “CI, not demented"; suspected dementia; probable dementia; assured syndrome of dementia).

### External classification

To ensure the standardization of the classification, an external expert (Prof. JF Dartigues) blindly checks the diagnosed cognitive status, subsequent to the full evaluation, by performing a review of the final diagnoses. In case of discordance, a panel consisting of the study team and the external expert discusses the final decision with a view to obtaining a consensus (Figure 2, Classification committee). The external classification leads to the validated diagnosis of “absence of CI”, “isolated cognitive complaint”, “CI without cognitive complaint”, “MCI”, “dementia”, and “other CI”.

### Biological analysis

Biological examinations are performed on the collected blood samples. The analyses carried out at the CHL are performed immediately after arrival of the samples: total cholesterol, high density lipoprotein (HDL-) and low-density lipoprotein (LDL-) cholesterol, triglycerides, thyroid stimulating hormone (TSH), homocysteine, folic acid and vitamin B12. In the *Life Sciences Research Unit,* the samples are prepared for further screening of the typical pro- and anti-inflammatory cytokines IL-1α, IL-1β, IL-4, IL-6, IL-8, IL-10, IL-13, MCP-1, IFNγ and TNFα. The rest of the analyses are carried out in the Laboratoire de Biochimie er Biologie Moléculaire, CHU, Nancy, France and are performed after a 6-month maximum storage period at -20°C. These analyses relate to the fatty acid profiles of red blood cell membrane phospholipids: (1) saturated fatty acids, such as palmitic (C16:0) and stearic (C18:0) acids; (2) mono-unsaturated fatty acids, respectively omega-7 and -9: palmitoleic (C16:1N-7) and oleic (C18:1N-9) acids and finally (3) poly-unsaturated fatty acids of omega-3 and omega-6 groups, including alpha-linolenic acid (ALA C18:3 n-3), eicosapentaenoic acid (EPA C20:5 n-3), docosahexaenoic acid (DHA C22:6 n-3) as well as linoleic acid (LA C18:2 n-6), arachidonic acid (ARA C20:4 n-6) and docosatetraenoic acid (C22:4 n-6). Moreover the vitamin B6 assay is also performed in Nancy to complete the picture of the blood concentrations of the “trio of vitamins B6, B9, B12” and the status of the homocysteinemia. Besides these overall analyses, APOE genetic polymorphism is investigated as well.

### Statistical analyses

Descriptive statistics based on means, standard deviations, percentages, odds ratios (for nested case-control studies) or relative risks (for cohort study) and 95% confidence intervals are used to depict the studied population. Potential confounding factors are investigated in view to include appropriate adjustments to statistical modeling. A careful attention is paid to possible biases. A logistic regression model is used to investigate association between MCI status and studied parameters. To this end, univariate analysis (Chi-square, Student t-test, or Mann-Withney test as appropriate) is carried out in order to select parameters linked to the outcome (p≤0.25). The model building is proceeded with stepwise backward elimination, requiring p < 0.05 for significance and starting with a model that contains all variables. After final selection, interactions between variables are tested following the same method. Clinically significant variables are forced into the model. The Wald chi-square test is used to assess the significance of variables in the model. The likelihood ratio test is calculated to estimate the effect of the deletion of a variable between subsequent models.

As for the cumulative incidence of participants with normal conditions, MCI or AD, both binomial and rates (with calculation of person-years in the cohort) are used. Relative risk (hazard ratio) of MCI and of evolution to AD are evaluated with the Cox proportional hazard model using a competing risks approach. Risk factors are further included in the model by using a model-building procedure. The results of neuropsychological testing are continuous data, and therefore, specific statistical procedures adapted to multivariate analysis of continuous data are used to assess changes over time.

The risk of MCI to AD conversion and the identification of risk factors for conversion between normal cognition, MCI and AD are estimated by using Markov processes.

A p-value of <0.05 is considered statistically significant. All tests are two-tailed. Statistical analyses are carried out with the statistical package SAS System version 9.2 (SAS Institute, Cary, North Carolina, USA).

### Quality

#### *Training for standardization of the team*

At two different levels of the study procedure, i.e. for the neuropsychological evaluation as well as for the neurogeriatric assessment, a group of 2 to 6 different investigators respectively perform the same exploration. Therefore, in addition to extremely strict and precise protocols, psychologists as well as neurologists and geriatricians attend standardization meetings at the beginning.

#### *Process control, traceability and validation of results*

Each step of collection, encoding or processing data and steps for preparing records of results are verified by two or even three people involved at specific stages of the control procedure. The encoding of the collected data is performed via an integral double entry.

### Follow-up procedure

A follow-up was included in the original design of the study. All the enrolled participants will yearly receive a phone call and a re-explanation of the study and its follow-up. Except the multilingualism assessment and the APOE screening, all people will again undergo the procedure of evaluation described in the protocol of this study.

### Ethics

The study is approved and authorized by the National Research Ethics Committee (CNER) and the National Commission for Data Protection (CNPD) in Luxembourg. Signed informed consent is obtained from all participants. The individual research results (neuropsychological test results, measures of blood pressure, heart rate, weight and height as well as blood test results) are returned to the participants and/or their general practitioner, if the participant mentioned it on the informed consent form.

## Discussion

The MemoVie study has been set up to depict the senior population of Luxembourg and to identify the conditions that could promote CI and evolution to AD. This study is planned to allow a long-term follow-up of the cognitive status of the population.

Several parameters are investigated, from health profiles and long-term intake of medications to occupational habits and socio-economic status. Particular attention is paid to multilingualism, as it constitutes one of the original aspects of our study. Among the major information generated by the MemoVie study, we aim to demonstrate that a set of various beneficial conditions are significantly linked to weak CI, which might confer a large panel of strategies to explore with a view to protecting memory. These conditions could be based on social interactions and particular habits, especially nutrition which is explored here through specific blood lipid components. This aspect could be essential in the setup of preventive interventions towards the senior population that exhibits moderate lipid disorders, thereby leading to higher risk of cardiovascular disease as well as cognitive decline. Indeed, the cardiovascular component of AD etiology could well be of high influence on the risk to develop the neurodegenerative disease.

Great amounts of data need to be analyzed in order to refine the results obtained, by including strategic adjustments, proposing models of several variables and studying the synergistic effects between the different conditions.

The results of the MemoVie study, more specifically focused on Luxembourg’s population, should provide insights to design new efficient approaches for community-level interventions intended to preserve overall health status and well-being in the global aging population.

## Endnotes

^a^The control group is constructed gradually to match with the group of persons suspected of showing CI, according to (i) age at enrollment, (ii) sex and (iii) education.

## Abbreviations

AD, Alzheimer’s disease; CERAD-NP, Consortium to establish a register for Alzheimer’s disease-neuropsychological battery; CI, Cognitive impairment; MCI, Mild cognitive impairment; CC, Cognitive complaint.

## Competing interests

The authors report no conflict of interest.

## Authors’ contributions

MP: drafting/revising of the manuscript, study concept or design, study supervision, classification committee; AMS: revising of the manuscript, study concept or design, neuropsychological evaluation supervision, classification committee; MV: drafting/revising of the manuscript, study concept or design, statistical methodology; ND: revising of the manuscript, study concept or design, Neurogeriatric assessment, classification committee AB: revising of the manuscript, study concept or design, Neurogeriatric assessment, classification committee; JCL: revising of the manuscript, study concept or design, Neurogeriatric assessment, classification committee; MDI: revising of the manuscript, research nurse evaluation, classification committee; JLL: revising of the manuscript, Neuropsychological evaluation, classification committee; DH: revising of the manuscript, Neuropsychological evaluation, classification committee; DU: revising of the manuscript, Neurogeriatric assessment, classification committee; ST: revising of the manuscript, Neurogeriatric assessment, classification committee; RD: revising of the manuscript, Neurogeriatric assessment, classification committee; PH: revising of the manuscript, study concept; SC: revising of the manuscript, study concept or design; JFD: revising of the manuscript, study concept or design; external expert of the classification committee; MLL: revising of the manuscript, study concept or design. All authors read and approved the final manuscript.

## Pre-publication history

The pre-publication history for this paper can be accessed here:

http://www.biomedcentral.com/1471-2458/12/519/prepub
